# Five-membered ring annelation in [2.2]paracyclophanes by aldol condensation

**DOI:** 10.3762/bjoc.10.210

**Published:** 2014-08-28

**Authors:** Henning Hopf, Swaminathan Vijay Narayanan, Peter G Jones

**Affiliations:** 1 Institut für Organische Chemie, Technische Universität Braunschweig, Hagenring 30, D-38106 Braunschweig, Germany, Fax: (+49) 531-391-5388; 2 Institut für Anorganische und Analytische Chemie, Technische Universität Braunschweig, Postfach 3329, D-38106 Braunschweig, Germany

**Keywords:** aldol condensation, cyclopentadienones, cyclophanes, multibridged cyclophanes, stereochemistry, X-ray analysis

## Abstract

Under basic conditions 4,5,12,13-tetraacetyl[2.2]paracyclophane (**9**) cyclizes by a double aldol condensation to provide the two aldols **12** and **15** in a 3:7 ratio. The structures of these compounds were obtained from X-ray structural analysis, spectroscopic data, and mechanistic considerations. On acid treatment **12** is dehydrated to a mixture of the condensed five-membered [2.2]paracyclophane derivatives **18**–**20**, whereas **15** yields a mixture of the isomeric cyclopentadienones **21**–**23**. The structures of these elimination products are also deduced from X-ray and spectroscopic data. The sequence presented here constitutes the simplest route so far to cyclophanes carrying an annelated five-membered ring.

## Introduction

**Scheme 1:**
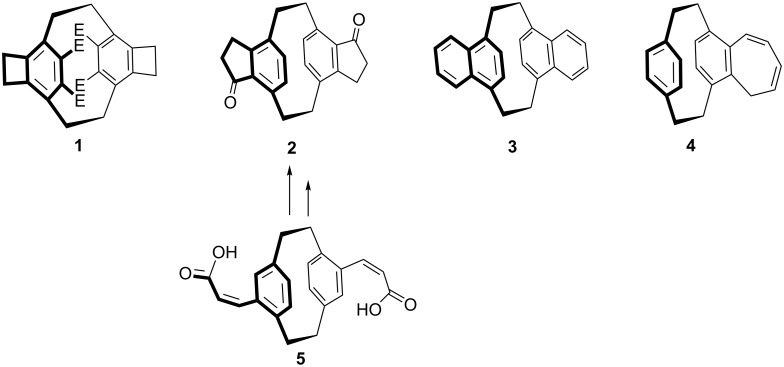
[2.2]Paracyclophane derivatives with annelated alicyclic rings.

We have reported in the past on numerous cyclophane derivatives bearing condensed four- to seven-membered rings, as shown by the representative examples of **1** (E = CO_2_CH_3_) [2], **2** [3], **3** [4], and **4** [5]. Many of these compounds possess interesting structural and spectroscopic properties, and can be used as substrates for novel cyclophanes (Scheme 1).

For example, **2** has been converted into the corresponding bis-olefin, from which multi-layered ferrocene complexes (“metallocenophanes”) were prepared [6]. Analogously, from **4** several tropyliophane salts were obtained [5].

In the case of the cyclopentenone-annelated compounds **2** we previously prepared precursors such as **5** (easily available by Wittig–Horner reaction of the appropriate bis-formyl cyclophanes) and subjected them to catalytic hydrogenation followed by a double Friedel–Crafts cyclization.

**Scheme 2:**
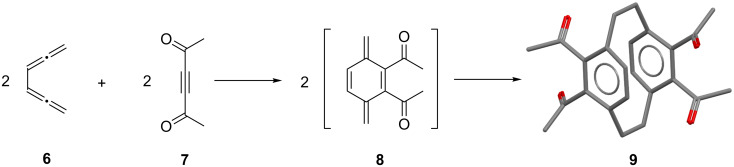
The formation of the tetraketone **9** by a Diels–Alder addition.

However, it appeared to us that a more direct route to these useful phane derivatives could be developed by employing aldol-type reactions of the tetraacetyl derivative **9**. This tetraketone (whose “twisted” structure was determined by single-crystal X-ray analysis [7] as shown in Scheme 2) is readily available by adding hex-3-yne-2,5-dione (**7**) to the diene hexa-1,2,4,5-tetraene (**6**), passing through the *p*-xylylene intermediate **8** *en route* [7].

**Scheme 3:**
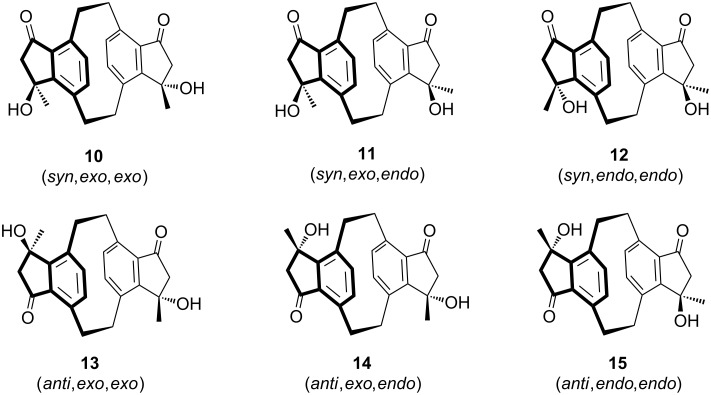
The possible structures of the aldols formed from **9**.

We were, furthermore, interested in studying the aldol reaction of **9**, since this process should pose several stereochemically interesting questions. As illustrated in Scheme 3, there are six different products that could be formed by an aldol cyclization of **9** (Scheme 3).

In the final product the two “surviving” carbonyl groups could point either in the same direction (*syn*-orientation, **10**–**12**) or in opposite directions (*anti*-orientation, **13**–**15**). Furthermore, the newly formed hydroxy group could either point towards the inner cavity of the cyclophane framework (*endo*-orientation) or to the outer section of the derivative (*exo*-orientation). Thus altogether the six different diastereomers **10**–**15** result.

## Results and Discussion

As the experiment shows (treatment of **9** with either sodium hydroxide in methanol or aniline in methanol, both at room temperature), only two of the possible six isomers (see above) are actually formed. To these we assign structures **12** and **15** (product ratio 30:70), i.e., the aldols are *syn*,*endo*,*endo*- and *anti*,*endo*,*endo*-isomers.

**Figure 1:**
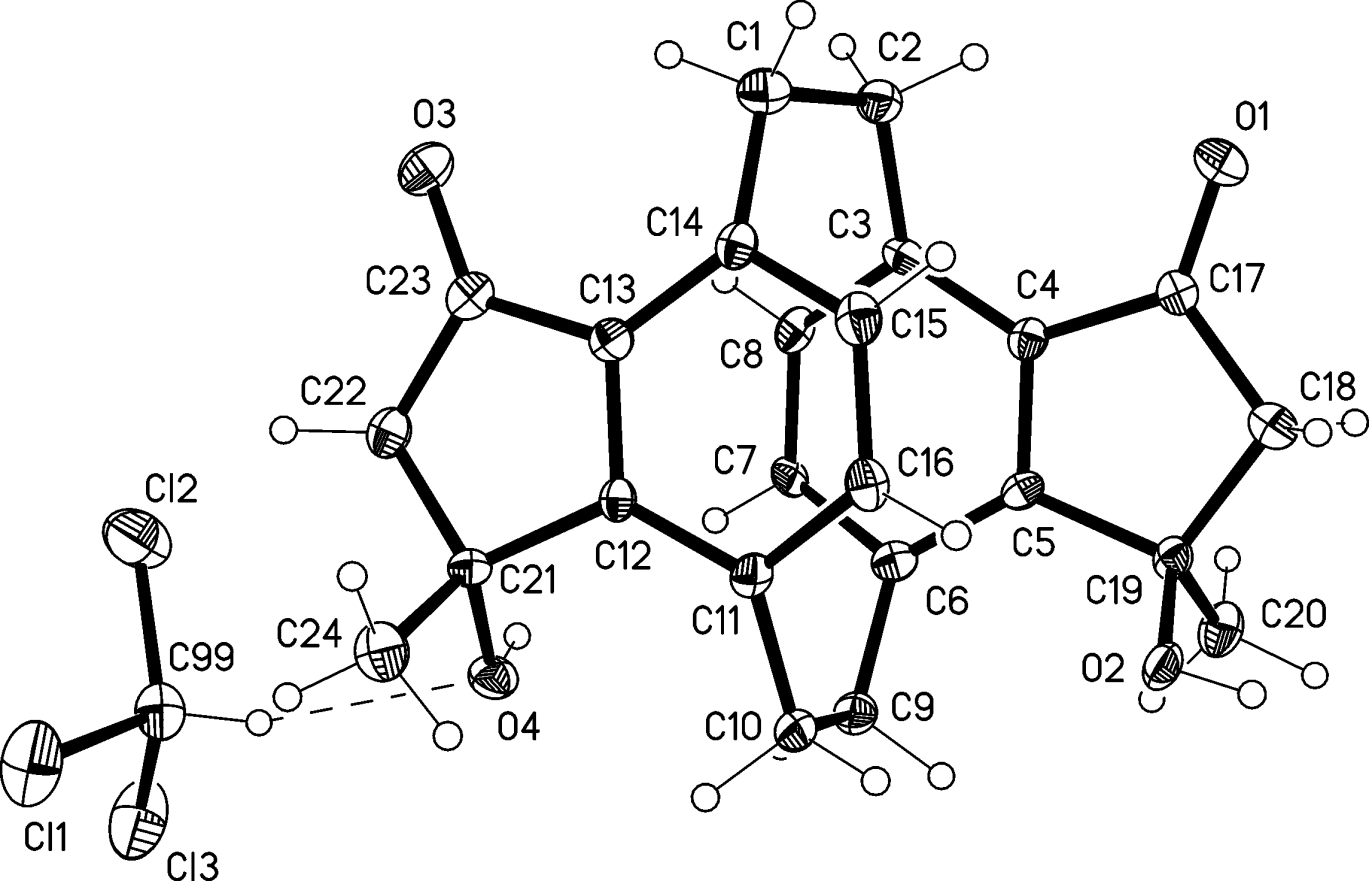
Structure of **12·**CDCl_3_ in the crystal. Ellipsoids represent 50% probability levels. Selected bond lengths (Å) and angles (°): C1–C2 1.573(11), C9–C10 1.579(11), C14–C1–C2 112.8(7), C1–C2–C3 112.1(6), C6–C9–C10 113.0(7), C9–C10–C11 112.1(7), C4–C3–C8 114.8(8), C5–C6–C7 115.3(7), C12–C11–C16 116.2(7), C13–C14–C15 115.1(8). The dashed line indicates a weak hydrogen bond.

For the former compound the structure assignment follows from single-crystal X-ray analysis of its deuteriochloroform solvate. The asymmetric unit is shown in Figure 1. The structure of **12** displays approximate *C*_2_ symmetry, with a r.m.s. deviation of 0.05 Å. It shows the normal distortions associated with the [2.2]paracyclophane geometry, with lengthened bridge bonds, widened sp^3^ bridge angles and narrowed sp^2^ ring angles at the bridgehead atoms, and boat-shaped six-membered rings with the bridgehead atoms lying out of the plane of the other four atoms (see Table 2 below). The crystal packing involves two classical and three "weak" hydrogen bonds (two involving the deuteriochloroform) that combine to form a complex three-dimensional pattern (Table 1).

**Table 1:** Hydrogen bonds [Å and °] for **12**^.^CDCl_3_.^a^

D-H···A	d(D-H)	d(H···A)	d(D···A)	<(DHA)

O(2)-H(02)···O(1)#1	0.84	1.98	2.791(8)	161.0
O(4)-H(04)···O(3)#2	0.84	2.13	2.917(8)	156.5
C(7)-H(7)···O(3)#2	0.95	2.36	3.307(10)	177.6
C(22)-H(22B)···Cl(3)#5	0.99	2.78	3.750(9)	167.7
C(99)-D(99)···O(4)	1.00	2.50	3.460(10)	161.2

^a^Symmetry transformations used to generate equivalent atoms: #1 −*x*+1,−*y*+2,*z−*1/2; #2 −*x*+1/2,*y*,*z−*1/2; #3 −*x*+1,−*y*+1,*z*+1/2; #4 *x*+1/2,−*y*+1,*z*; #5 −*x*+1/2,*y*,*z*+1/2.

Several attempts to obtain crystals of the major isomer **15** suitable for X-ray analysis failed. The *anti*-orientation of the carbonyl groups of compound **15** was proved by the X-ray structural analysis of the product resulting from a dehydration experiment described below.

The most plausible mechanism for the above aldol reaction also favors the discussed structural assignments. As shown above in Scheme 2, the starting material **9** favors a conformation in the solid state in which all carbonyl groups point towards the “inner” space of the cyclophane core. In other words, the oxygen atom faces a methylene hydrogen atom of the bridge. In principle the alternative orientation is also possible, with the methyl substituent taking this position. A comparable situation has been observed for the 4-acetyl derivative of [2.2]paracyclophane, with the difference that the preferred *endo*-conformation has been observed both for the solid state [8–10] and in solution by NMR spectroscopy [11].

**Scheme 4:**
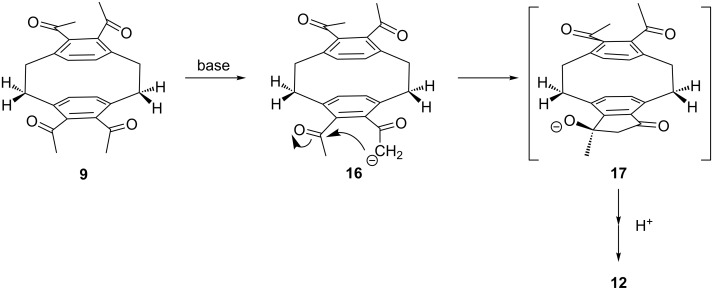
The mechanism of the aldol cyclization.

We hence assume that the conformation of **9** in solution resembles that of the solid state structure given in Scheme 2. With this assumption we can easily rationalize the formation of **12** and **15** in the aldol reaction, both containing *endo*-configurated OH groups only (Scheme 4).

The reaction is initiated by proton abstraction from one of the acetyl groups (**9**→**16**). The resulting enolate subsequently attacks the vicinal acetyl group, pushing the developing alcoholate function towards the methylene group, while the methyl substituent stays at the “outside” of the layered system. The intermediate **17** thus generated is then protonated to the corresponding aldol. Repetition of the whole process at the other deck of the cyclophane finally produces **12**.

Presumably the same mechanism is followed during the formation of the main product **15**, the only difference between the pathways being the enolate formation, i.e., whether the second five-membered ring is produced by deprotonating a *syn*- or *anti*-positioned acetyl group.

To learn more about the chemical properties of the above aldols, we next dehydrated them under acidic conditions.

**Scheme 5:**
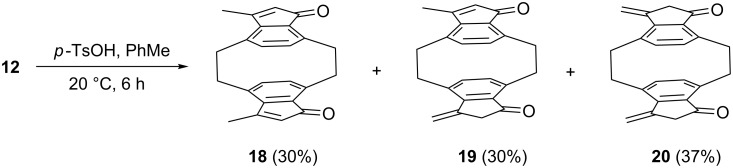
Dehydration of the aldol **12**.

When the *syn*-isomer **12** was treated with *p*-toluenesulfonic acid in toluene at room temperature, three dehydration products **18**–**20** were produced in approximately equal yields. The products were separated by preparative plate chromatography, and the conjugated derivatives **18** and **19** display a characteristic bright yellow color and show absorption maxima at 363 and 350 nm in the UV–vis spectrum, respectively. The unconjugated isomer **20** is a colorless solid and possesses a UV maximum at 247 nm. IR and NMR spectra (see Supporting Information File 1) support these structure assignments (Scheme 5).

It is interesting to note that for the formation of all three isomers an inwardly-oriented OH group of **12** has to be attacked; evidently there is enough room at this part of the substrate to enable protonation. The subsequent proton loss from the intermediately generated carbocation can proceed either by a Hofmann or a Saytseff mode, and – as the results show – both pathways are followed. Stereoelectronic reasons are presumably responsible for the slight preference of the Hofmann route. Not only does the methyl group appear to be sterically preferred for proton removal, it is also statistically favored. Furthermore, the newly produced cyclopentadienone ring possesses anti-aromatic character and should hence be disfavored as compared to the non-conjugated methylencyclopentenone ring in **19** or **20**.

**Scheme 6:**
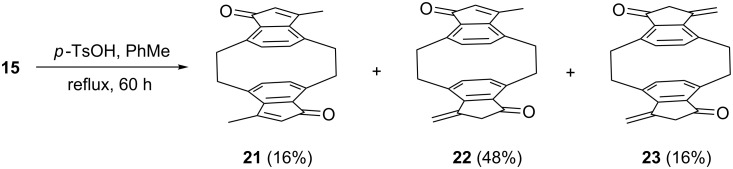
Dehydration of the aldol **15**.

The *anti*-isomer **15** loses water under somewhat harsher conditions, and also furnishes three dehydration products, the *anti*-configurated diketones **21**–**23**, produced in a 1:3:1 ratio. Again the fully or partially conjugated isomers are deeply colored (intense yellow) and display absorption maxima at 389 and 358 nm, respectively (Scheme 6).

**Figure 2:**
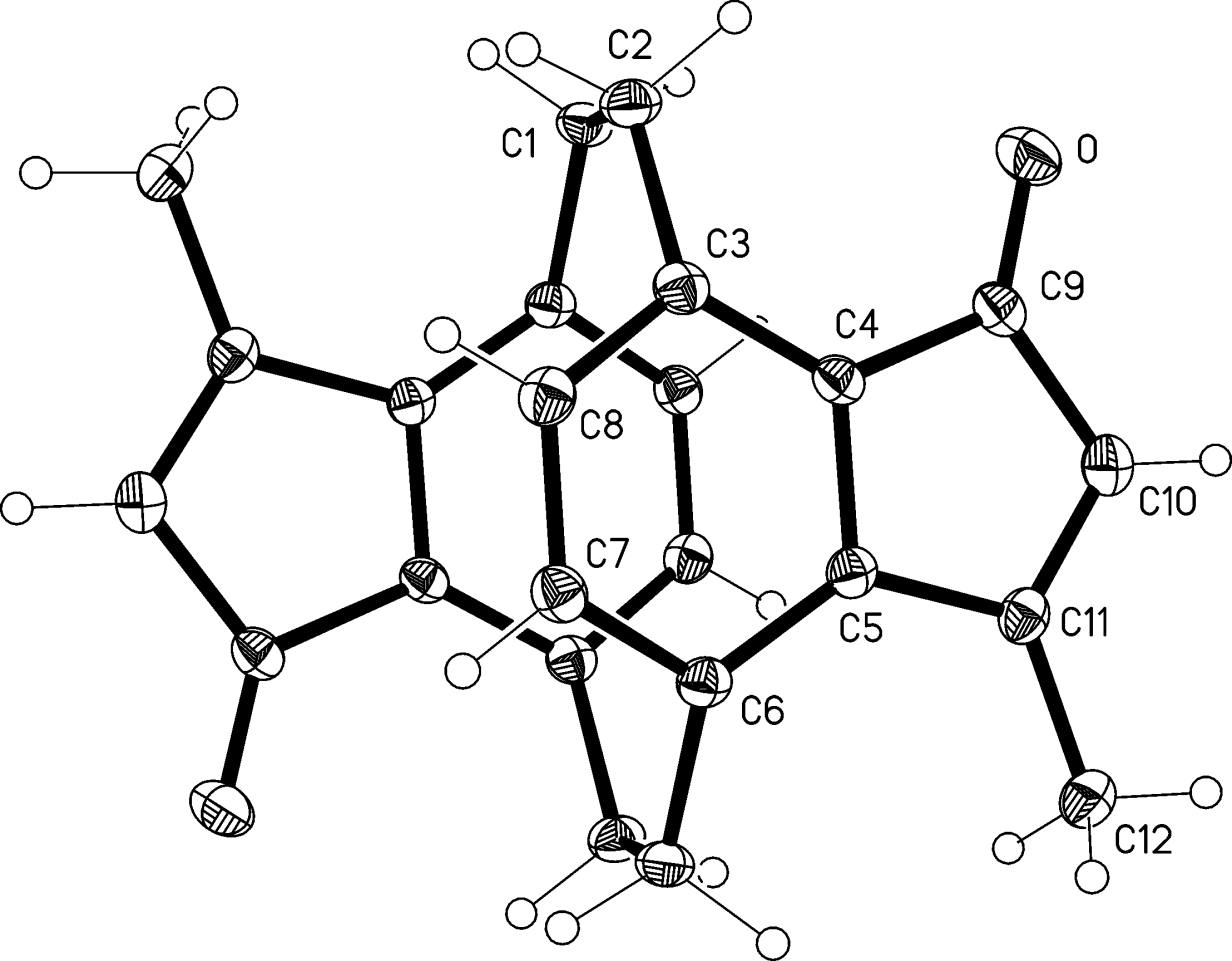
Structure of compound **21** in the crystal. Ellipsoids represent 50% probability levels. Selected bond lengths (Å) and angles (°): C1–C2 1.589(2), C6'–C1–C2 112.9(1), C1–C2–C3 111.8(1), C4–C3–C8 115.3(1), C5–C6–C7 115.4(1).

For **21** yellow single crystals were obtained by recrystallization from pentane/chloroform to allow their X-ray structure determination in the solid state. The results are shown in Figure 2. The molecule displays crystallographic inversion symmetry (which precludes the use of standard cyclophane numbering to some extent). It shows the same type of distortions as discussed above for **12** (see Table 2).

**Table 2:** Crystallographic data for **12**^.^CDCl_3_ and **21**.

Compound	**12**^.^CDCl_3_	**21**

Formula	C_25_H_24_DCl_3_O_4_	C_24_H_20_O_2_
*M*_r_	496.81	340.40
Habit	colourless plate	yellow tablet
Cryst. size (mm)	0.3 × 0.12 × 0.02	0.4 × 0.2 × 0.08
Crystal system	orthorhombic	monoclinic
Space group	*Pca*2_1_	*C*2/*c*
Temperature (°C)	–140	–140
Cell constants:		
*a* (Å)	19.286(3)	14.051(2)
*b* (Å)	12.253(2)	8.0225(12)
*c* (Å)	9.527(2)	14.536(2)
α (°)	90	90
β (°)	90	96.695(6)
γ (°)	90	90
*V* (Å^3^)	2251.2	1627.5
*Z*	4	4
*D*_x_ (Mg m^-3^)	1.466	1.389
μ (mm^-1^)	0.44	0.09
*F*(000)	1032	720
2θ_max_	52.6	60
Refl. measured	8580	9043
Refl. indep.	3249	2394
*R*_int_	0.112	0.081
Parameters	413	119
Restraints	293	0
*wR*(*F*^2^, all refl.)	0.165	0.140
*R*(*F*, >4σ(*F*))	0.091	0.051
*S*	1.19	1.06
max. Δρ (e Å^-3^)	0.43	0.45

This result clearly confirms our previous structure assignment of **15** as an *anti*-isomer. Although structures **13** and **14** for the initial aldol products cannot be excluded from the dehydration experiment, this alternative seems unlikely since it would require an aldol-formation step different from that discussed above (Scheme 4), i.e., one in which (several) of the carbonyl groups would point away from the ethano bridge. Based on the above structural results, there is no reason to assume this conformation to be preferred in the present case.

## Conclusion

In summary, we have demonstrated in this study that aldol-type condensations constitute an attractive alternative to prepare [2.2]paracyclophane derivatives with annelated (and functionalized) five-membered rings, making these derivatives easily available for further transformations.

## Supporting Information

File 1 Experimental part.
