# Supplementary material for: Five-membered ring annelation in [2.2]paracyclophanes by aldol condensation
Source: Beilstein J Org Chem. 2014 Aug 28;10:2021–6. doi: 10.3762/bjoc.10.210 (PMC4168897; doi:10.3762/bjoc.10.210)
Supplement: File 1 — Experimental part. [file Beilstein_J_Org_Chem-10-2021-s001.pdf]

Supporting Information

for

Five-membered ring annelation in [2.2]paracyclophanes by aldol condensation

Henning Hopf*[1], Swaminathan Vijay Narayanan[1] and Peter G. Jones[2]

Address: [1]Institut für Organische Chemie, Technische Universität Braunschweig, Hagenring 30, D-38106 Braunschweig, Germany, Fax: (+49) 531-391-5388 and [2]Institut für Anorganische und Analytische Chemie, Technische Universität Braunschweig, Postfach 3329, D-38106 Braunschweig, Germany

Email: Henning Hopf - h.hopf@tu-bs.de

Peter G. Jones - p.jones@tu-bs.de

* Corresponding author

Experimental part

Experimental section:

General: Chromatography: TLC: precoated plastic plates, Silica gel (SiO_2): Polygram Sil G/UV_{254} (Macherey-Nagel & Co.) or on Alumina (Al_2O_3): Polygram ALOX N/UV_{254} (Macherey-Nagel & Co.); CC: Column chromatography was performed on Silica gel 60 (70-230 mesh), Merck (Darmstadt). – Melting points: Büchi 510 melting point apparatus, MEL-TEMP II (Laboratory Devices, USA) or Kofler heated stage microscope; the melting points are uncorrected. - **NMR spectroscopy:** ^{1}H and ^{13}C spectra were recorded on a Bruker AC-200: ^{1}H NMR (200.1 MHz); ^{13}C NMR (50.3 MHz) and a Bruker DRX-400: ^{1}H NMR (400.1 MHz); ^{13}C NMR (100.6 MHz). Deuteriochloroform ($CDCl_3$) was used as solvent unless otherwise stated. Chemical shifts are reported in parts per million (δ) downfield from the internal tetramethylsilane reference and the coupling constants are in Hertz. - **IR spectroscopy:** Nicolet 320 FT-IR or Bruker Tensor 27 spectrometer. Samples were prepared as KBr pellets or as thin films. - **UV/Vis spectroscopy:** Varian Cary 100BIO or HP 8452A Diode Array spectrophotometer. - **MS:** Finnigan MAT 95 spectrometer using electron ionisation (EI, 70 eV); ESI spectra: Finnigan MAT 95XLT spectrometer. Spray voltage 1.3-1.8 kV, flow rate 0.5-1.5 µL per min. Methanol was used as solvent unless otherwise specified. - **GC/MS:** Finnigan TSQ 700 (EI, 70 eV) attached to a Hewlett Packard 5890A gas chromatograph. - Nitrogen and argon gas were used for maintaining an inert atmosphere. All solvents and chemicals were purified as prescribed in

Purification of Laboratory Chemicals, 4th edition, Butterworth-Heinemann, Oxford 2000.

Aldol reaction of 4,5,12,13-tetraacetyl[2.2]paracyclophane (9): (a) A dried, N_2-flushed Schlenk flask was charged with 0.40 g of NaOH and anhydrous methanol (10 mL) and the mixture was cooled in an ice/water bath with stirring. After all the NaOH had dissolved, the bath was removed, 4,5,12,13-tetraacetyl[2.2]paracylophane (**9**, 100 mg, 0.267 mmol) was added in one portion and the mixture was stirred at room temperature. During 15 min the color turned to turbid yellow, then to brown, and at the end to reddish brown. The reaction mixture was stirred at room temperature for 24 h, and was then quenched by adding water followed by dichloromethane. One of the products, namely the *anti*-isomer **15**, precipitated; it was removed by filtration and dried yielding a pale brown solid (70 mg, 70%). The rest of the filtrate was extracted with dichloromethane. The organic phase was washed several times with water, brine and finally dried with anhydrous $MgSO_4$; the solvent was removed by rotary evaporation yielding the *syn*-isomer **12**, also as a pale brown solid (30 mg, 30%). Compound **12** was recrystallized from deuteriochloroform to furnish plate-like crystals used for X-ray structural analysis (see below). - (b) A N_2-flushed 25 mL round-bottomed flask was charged with 4,5,12,13-tetraacetyl[2.2]paracyclophane (**9**, 100 mg, 0.267 mmol) along with anhydrous methanol (10 mL) and aniline (2.28 g, 26.59 mmol) with stirring at room temperature. The stirring was continued at

the same temperature for 8 d avoiding exposure to light. For workup the solvent was evaporated under reduced pressure followed by addition of diethyl ether. The precipitate formed was filtered off and was found to be the *anti*-isomer **15** (40 mg, 40%) as a brown solid. The filtrate was washed with 2 N aq. HCl, sat. aq. $NaHCO_3$ solution, brine, and then dried ($MgSO_4$). The solvent was removed by rotary evaporation. The crude product was subjected to flash column chromatography on silica gel with 1:1 EtOAc/hexane as eluent, furnishing the *syn*-isomer **12** (20 mg, 20%) as a colorless solid.

***syn*(*endo*,*endo*)-19,21-Dihydroxy-19,21-dimethyl[2.2]indanonophane-17,23-dione (12):** m.p.: >255 °C (decomp.)- **^{1}H NMR** (400.1 MHz, $CDCl_3$): δ = 6.92 (d, 2 H, J = 7.63 Hz, Ar-H), 6.36 (d, 2 H, J = 7.62 Hz, Ar-H), 4.13 – 4.05 (m, 2 H, bridge-H), 3.62 – 3.54 (m, 2 H, bridge-H), 3.13 – 3.07 (m, 2 H, bridge-H), 2.98 – 2.91 (m, 2 H, bridge-H), 2.82 (d, 2 H, J_{gem} = 18.32 Hz, 18-H, 22-H), 2.68 (d, 2 H, J_{gem} = 18.30 Hz, 18-H, 22-H), 1.99 (br. s, 2 H, OH), 1.47 ppm (s, 6 H, 20-H, 24-H). - **^{13}C NMR** (100.6 MHz, $CDCl_3$): δ = 203.1 (s, C-17, C-23), 155.7 (s, Ar), 140.7 (s, Ar), 140.6 (d, Ar), 137.2 (s, Ar), 136.5 (s, Ar), 133.5 (d, Ar), 74.9 (s, C-19, C-21), 54.6 (t, C-18, C-22), 33.0 (t, bridge), 32.2 (q, C-20, C-24), 30.2 ppm (t, bridge). - **IR** (diamond-ATR): nu(tilde) = 3479 (m), 2975 (w), 2887 (w), 1668 (vs), 1559 (m), 1484 (w), 1450 (w), 1367 (w), 1244 (m), 1061 (m), 1040 (w), 954 (w), 873 (w), 603 cm^{-1}. - **UV/Vis** (CH_2Cl_2): λ_{max} (lg ε) = 330 (2.87), 288 nm (3.65). - **MS** (EI, 70 eV): *m/z* (%) = 376 (1) [M^+], 358 (39), 340

(53), 325 (15), 312 (10), 297 (13), 187 (12), 170 (100), 142 (36), 141 (35), 128 (10), 115 (25), 91 (8), 77 (5), 63 (5), 51 (5), 40 (5). - **HRMS** ($C_{24}H_{23}O_4$) $[M-H]^+$ calcd.: 375.1596; found: 375.15942. - **Elemental analysis**: calcd.: C 76.57, H 6.43; found: C 75.60, H 6.20. Note that **12** forms solvates (see X-ray).

***anti*(*endo*,*endo*)-19,23-Dihydroxy-19,23-dimethyl[2.2]indanonophane-17,21-dione (15)**: **m.p.**: >380 °C. - **^{1}H NMR** (400.1 MHz, DMSO-d_6): δ = 6.85 (d, 2 H, ^3J = 7.57 Hz, Ar-H), 6.43 (d, 2 H, ^3J =7.59 Hz, Ar-H), 5.61 (br.s, 2 H, OH), 4.00 – 3.95 (m, 2 H, bridge-H), 3.76 – 3.70 (m, 2 H, bridge-H), 3.05 – 2.96 (m, 2 H, bridge-H), 2.92 – 2.88 (m, 2 H, bridge-H), 2.84 (d, 2 H, ^2J = 18.25 Hz, 18-H, 22-H), 2.63 (d, 2 H, ^2J = 18.21 Hz, 18-H, 22-H), 1.36 ppm (s, 6 H, 20-H, 24-H). - **^{13}C NMR** (100.6 MHz, DMSO-d_6): δ = 203.5 (s, C-17, C-21), 157.5 (s, C-19, C-23), 139.3 (s, Ar), 138.7 (d, Ar), 136.7 (s, Ar), 135.4 (s, Ar), 134.6 (d, Ar), 133.0 (s, Ar), 54.3 (t, C-18, C-22), 31.7 (q, C-20, C-24), 31.1, 30.3 ppm (t, C-1, C-2, C-9, C-10). - **IR** (diamond-ATR): nu(tilde) = 3428 (w), 2960 (w), 2983 (w), 1674 (s), 1588 (w), 1558 (w), 1250 (w), 1113 (w), 1067 (w), 870 (w), 604 cm^{-1} (w). - **UV/Vis** (MeOH): λ_{max} (lg ε) = 292 nm (3.06). - **MS** (EI, 70 eV): *m/z* (%) = 376 (2) [M^+], 358 (20), 340 (44), 325 (15), 312 (17), 297 (16), 170 (100), 141 (52), 115 (28), 93 (34), 77 (6), 63 (6), 42 (4). - **HRMS** ($C_{24}H_{24}O_4$) calcd.: 376.16748; found: 376.16628 ± 1 ppm. - **Elemental analysis**: calcd.: C 76.57, H 6.43; found: C 75.06, H 6.30. Note that compounds of this type form solvates (see above).

Dehydration reaction of 12: A dried, N_2-flushed round-bottomed flask was charged with **12** (30 mg, 0.0798 mmol) along with anhydrous toluene (15 mL) and a pinch of *p*-TsOH. The mixture was stirred at room temperature for 6 h, the solvent was removed under reduced pressure, and the crude product was subjected to preparative TLC on silica gel with 1% EtOAc in CH_2Cl_2, resulting in three isomers: derivative **18** (8.1 mg, 30%), yellow solid; **19** (8.2 mg, 30%), yellow solid; **20** (0.01 g, 37%), colorless solid.

***syn*-19,21-Dimethyl[2.2]indenonophane-17,23-dione (18): m.p.:** 150 °C. - **^{1}H NMR** (400.1 MHz, $CDCl_3$): δ = 6.39 (d, 2 H, *J* = 8.07 Hz, Ar-CH), 6.22 (d, 2 H, *J* = 8.06 Hz, Ar-CH), 5.61 (q, 2 H, *J* = 1.57 Hz, 18-H, 22-H), 3.95 – 3.88 (m, 2 H, bridge-H), 3.48 – 3.40 (m, 2 H, bridge-H), 2.75 – 2.62 (m, 4 H, bridge-H), 2.30 ppm (d, 6 H, *J* = 1.58 Hz, 20-H, 24-H). - **^{13}C NMR** (100.6 MHz, $CDCl_3$): δ = 203.8 (s, C-17, C-23), 167.4 (s, C-19, C-21), 140.5 (s, Ar), 136.5 (d, Ar), 134.5 (s, Ar), 133.4 (d, Ar), 125.7 (d, C-18, C-22), 33.4 (t, bridge), 29.2 (t, bridge), 17.2 ppm (q, C-20, C-24); two aromatic quaternary carbons were not detected because of low intensity. - **IR** (diamond-ATR): nu(tilde) = 2922 (m), 2852 (w), 1688 (vs), 1562 (m), 1430 (m), 1251 (w), 1214 (w), 1051 (s), 827 (m), 795 (m), 626 cm^{-1} (m). - **UV/Vis** (CH_3CN): λ_{max} (lg ε) = 363 (3.09), 252 nm (4.17). - **MS** (EI, 70 eV): *m/z* (%) = 340 (100) [M^+], 325 (17), 312 (12), 297 (20), 269 (7), 170 (100), 141 (44), 115 (16), 43 (8). - **HRMS** ($C_{24}H_{20}O_2$) calcd.: 340.14633; found: 340.14509.

***syn*-19-Methyl-21-methylene[2.2]indano-18-enophane-17,23-dione (19): m.p.:** 195 °C. - ^{1}**H NMR** (400.1 MHz, $CDCl_3$): δ = 6.77 (d, 1 H, *J* = 7.59 Hz, Ar-H), 6.44 (d, 1 H, *J* = 7.59 Hz, Ar-H), 6.39 (d, 1 H, *J* = 8.09 Hz, Ar-H), 6.12 (d, 1 H, *J* = 8.09 Hz, Ar-H), 5.78 (t, 1 H, *J* = 1.83 Hz, 24-H), 5.60 (q, 1 H, *J* = 1.59 Hz, 18-H), 5.39 (t, 1 H, *J* = 1.55 Hz, 24-H), 4.11 – 4.06 (m, 1 H, bridge-H), 3.88 – 3.77 (m, 2 H, bridge-H), 3.35 – 3.29 (m, 1 H, bridge-H), 3.12 (s, 2 H, 22-H), 3.09 – 2.97 (m, 1 H, bridge-H), 2.89 – 2.71 (m, 3 H, bridge-H), 2.30 ppm (d, 3 H, *J* = 1.65 Hz, 20-H). - ^{13}**C NMR** (100.6 MHz, $CDCl_3$): δ = 203.3 (s, C-17, C-23), 162.8 (s, C-19), 151.5 (s, C-21), 146.7 (s, Ar), 141.5 (s, Ar), 140.4 (s, Ar), 138.2 (s, Ar), 138.1 (s, Ar), 137.7 (d, Ar), 137.0 (s, Ar), 136.4 (d, Ar), 135.0 (s, Ar), 134.9 (d, Ar), 133.1 (s, Ar), 132.4 (d, Ar), 125.9 (d, C-18), 111.7 (t, C-24), 43.6 (t, C-22), 43.5 (t, C-1, C-2), 35.54 (t, bridge), 35.55 (t, bridge), 30.4 ppm (q, C-20). - **IR** (diamond-ATR): nu(tilde) = 2930 (w), 2851 (w), 1695 (s), 1556 (m), 1428 (w), 1391 (w), 1259 (m), 1214 (w), 1096 (w), 1049 (m), 1011 (m), 866 (m), 801 (m), 784 (m), 621 cm^{-1} (w). - **UV/Vis** (CH_3CN): λ_{max} (lg ε) = 350 (3.27), 249 nm (4.35). - **MS** (EI, 70 eV): *m/z* (%) = 340 (67) [M^+], 322 (16), 297 (10), 170 (100), 141 (44), 115 (17), 43 (5). - **HRMS** ($C_{24}H_{20}O_2$) calcd.: 340.14633; found: 340.14536.

***syn*-19,21-Dimethylene[2.2]indanonophane-17,23-dione (20): m.p.:** >195 °C (decomp.). - ^{1}**H NMR** (400.1 MHz, $CDCl_3$): δ = 6.70 (d, 2 H, *J* = 7.61 Hz, Ar-H), 6.34 (d, 2 H, *J* = 7.56 Hz, Ar-H), 5.79 (s, 2 H, 20-H, 24-H), 5.39 (s, 2 H, 20-H, 24-H), 4.08 – 4.01 (m,

2 H, bridge-H), 3.69 – 3.63 (m, 2 H, bridge-H), 3.08 (d, 4 H, *J* = 1.81 Hz, 18-H, 22-H), 2.92 – 2.85 ppm (m, 2 H, bridge-H). - ^{13}**C NMR** (100.6 MHz, $CDCl_3$): δ = 202.6 (s, C-17, C-23), 151.7 (s, C-19, C-21), 140.8 (s, Ar), 140.6 (s, Ar), 139.9 (s, Ar), 136.5 (d, Ar), 135.9 (s, Ar), 132.0 (d, Ar), 111.4 (t, C-20, C-24), 42.9 (t, C-18, C-22), 31.4 (t, bridge), 30.1 ppm (t, bridge). - **IR** (diamond-ATR): nu(tilde) = 2923 (m), 2852 (w), 1692 (vs), 1554 (w), 1459 (w), 1445 (w), 1379 (w), 1315 (w), 1252 (s), 1042 (m), 866 (m), 782 (m), 610 cm^{-1} (m). - **UV/Vis** (CH_3CN): λ_{max} (lg ε) = 247 nm (4.30). - **MS** (EI, 70 eV): *m/z* (%) = 340 (64) [M^+], 322 (24), 312 (12), 170 (100), 141 (48), 115 (18), 57 (10), 41 (4). - **HRMS** ($C_{24}H_{20}O_2$) calcd.: 340.14633; found: 340.14556.

Dehydration of 15: A dried N_2-flushed Schlenk flask with reflux condenser and a stirrer was charged with **15** (70 mg, 0.187 mmol) along with anhydrous toluene (25 mL) and a pinch of *p*-TsOH. The reaction mixture was refluxed with stirring for 60 h, the mixture was cooled to room temp., and the solvent was evaporated under reduced pressure. The crude product mixture was subjected to preparative TLC on silica gel with 17:3 CH_2Cl_2/pentane resulting in three isomers: derivative **21** (10 mg, 16%), yellow crystalline solid, **22** (30 mg, 47%), yellow solid, **23** (10 mg, 16%), colorless solid. Compound **21** was recrystallized from CH_2Cl_2/pentane by the slow diffusion method to furnish yellow tablet-shaped crystals used for X-ray analysis (see below).

***anti*-19,23-Dimethyl[2.2]indenonophane-17,21-dione (21): m.p.:** 253 °C. - ^{1}**H NMR** (400.1 MHz, $CDCl_3$): δ = 6.36 (d, 2 H, *J* = 8.05

Hz, 8-H, 16-H), 6.26 (d, 2 H, *J* = 8.05 Hz, 7-H, 15-H), 5.60 (q, 2 H, *J* = 1.43 Hz, 18-H, 22-H), 4.01 – 3.96 (m, 2 H, 2-H, 10-H), 3.36 – 3.29 (m, 2 H, 1-H, 9-H), 2.94 – 2.86 (m, 2 H, 1-H, 9-H), 2.56 – 2.49 (m, 2 H, 2-H, 10-H), 2.32 ppm (d, 6 H, *J* = 1.33 Hz, 20-H, 24-H). - ^{13}**C NMR** (100.6 MHz, $CDCl_3$): δ = 198.4 (s, C-17, C-21), 163.1 (s, C-19, C-23), 145.7 (s, C-5, C-13), 139.0 (s, C-3, C-11), 136.8 (d, C-7, C-15), 135.9 (s, C-6, C-14), 133.2 (d, C-8, C-16), 132.2 (s, C-4, C-12), 125.3 (d, C-18, C-22), 31.3 (t, C-2, C-10), 31.1 (t, C-1, C-9), 17.3 ppm (q, C-20, C-24). - **IR** (diamond-ATR): nu(tilde) = 2947 (w), 2928 (w), 1687 (vs), 1585 (w), 1558 (m), 1439 (w), 1424 (w), 1395 (w), 1271 (m), 1213 (w), 1029 (m), 1006 (m), 846 (s), 759 (m), 731 (m), 661 cm^{-1} (m). - **UV/Vis** (CH_3CN): λ_{max} (lg ε) = 389 (3.19), 356 (3.36), 251 nm (4.40). - **MS** (EI, 70 eV): *m/z* (%) = 340 (59) [M^+], 325 (10), 312 (33), 297 (24), 170 (100), 141 (47), 115 (18), 57 (22), 43 (4). - **HRMS** ($C_{24}H_{20}O_2$) calcd.: 340.14633; found: 340.14549.

***anti*-19-Methyl-23-methylene[2.2]indano-18-enophane-17,21-dione (22): m.p.**: >210 °C (decomp.). - ^{1}**H NMR** (400.1 MHz, $CDCl_3$): δ = 6.61 (d, 1 H, *J* = 7.45 Hz, 15-/16-H), 6.58 (d, 1 H, *J* = 7.63 Hz, 15-/16-H), 6.32 (d, 1 H, *J* = 8.07 Hz, 8-H), 6.16 (d, 1 H, *J* = 8.08 Hz, 7-H), 5.84 (t, 1 H, *J* = 1.6 Hz, 24-H), 5.59 (q, 1 H, *J* = 1.5 Hz, 18-H), 5.38 (t, 1 H, *J* = 1.39 Hz, 24-H), 4.21 – 4.14 (m, 1 H, 1-/10-H), 3.85 – 3.79 (m, 1 H, 2-H), 3.71 – 3.65 (m, 1 H, 1-/10-H), 3.35 – 3.29 (m, 1 H, 9-H), 3.11 (t, 2 H, *J* = 1.43 Hz, 22-H), 3.06 – 3.01 (m, 1 H, 1-/10-H), 2.94 – 2.81 (m, 2 H, 2-H, 9-H), 2.66 – 2.62 (m, 1 H, 1-/10-H), 2.30 ppm (d, 3 H, *J* =

1.49 Hz, 20-H). - ^{13}**C NMR** (100.6 MHz, $CDCl_3$): δ = 203.2 (s, C-17, C-21), 167.1 (s, C-12), 163.2 (s, C-19), 151.2 (s, C-13), 145.9 (s, C-5), 140.7 (s, C-23), 139.4 (s, C-11), 138.9 (s, C-3), 137.7 (d, C-15/-16), 137.6 (s, C-14), 136.5 (d, C-7), 135.6 (s, C-6), 132.5 (s, C-4), 132.2 (d, C-15/-16), 131.9 (d, C-8), 125.3 (d, C-18), 111.4 (t, C-24), 43.2 (t, C-22), 32.4 (t, C-1, C-10), 31.1 (t, C-9), 28.8 (t, C-2), 17.1 ppm (q, C-20). - **IR** (diamond-ATR): nu(tilde) = 2926 (w), 2852 (w), 1694 (s), 1555 (w), 1393 (w), 1261 (w), 1214 (w), 1050 (w), 1030 (w), 861 (w), 728 (w), 620 cm^{-1} (w). - **UV/Vis** (CH_3CN): λ_{max} (lg ε) = 358 (3.25), 252 nm (4.35). - **MS** (EI, 70 eV): *m/z* (%) = 340 (50) [M^+], 312 (12), 297 (12), 170 (100), 141 (40), 115 (19), 44 (16). - **HRMS** ($C_{24}H_{20}O_2$) calcd.: 340.14633; found: 340.14527.

***anti*-19,23-Dimethylene[2.2]indenonophane-17,21-dione (23)**: **m.p.**: >260 °C (decomp.). - ^{1}**H NMR** (400.1 MHz, $CDCl_3$): δ = 6.62 (d, 2 H, *J* = 7.59 Hz, Ar-H), 6.55 (d, 2 H, *J* = 7.58 Hz, Ar-H), 5.89 (s, 2 H, 20-H, 24-H), 5.46 (s, 2 H, 20-H, 24-H), 4.11 – 4.06 (m, 2 H, bridge-H), 3.77 – 3.72 (m, 2 H, bridge-H), 3.15 (s, 4 H, 18-H, 22-H), 3.13 – 3.03 ppm (m, 4 H, bridge-H). - ^{13}**C NMR** (100.6 MHz, $CDCl_3$): δ = 202.7 (s, C-17, C-21), 151.4 (s, C-19, C-23), 140.7 (s, Ar), 140.2 (s, Ar), 139.2 (s, Ar), 137.6 (d, Ar), 137.1 (s, Ar), 131.1 (d, Ar), 111.5 (t, C-20, C-24), 43.0 (t, C-18, C-22), 32.5 (t, bridge), 29.4 ppm (t, bridge). - **IR** (diamond-ATR): nu(tilde) = 2925 (m), 2853 (w), 1693 (s), 1553 (w), 1456 (w), 1262 (w), 1239 (w), 1044 (w), 890 (w), 783 (w), 603 cm^{-1} (w). - **UV/Vis** (CH_3CN): λ_{max} (lg ε) = 343 (3.44), 242 nm

(4.50). - **MS** (EI, 70 eV): *m/z* (%) = 340 (52) [M^+], 322 (15), 312 (9), 170 (100), 141 (50), 115 (17), 97 (4), 71 (5), 57 (8), 49 (4). - **HRMS** ($C_{24}H_{20}O_2$) calcd.: 340.14633; found: 340.14513.

X-Ray structure determinations

Crystals were mounted in inert oil on glass fibres and transferred to the cold gas stream of a Bruker SMART 1000 CCD diffractometer. Intensity data were recorded using monochromated Mo $K\alpha$ radiation (λ = 0.71073 Å). No absorption corrections were implemented. The structures were refined anisotropically on F^2 using the program SHELXL-97 [1]. Methyl groups were refined as idealized rigid groups allowed to rotate but not tip, OH hydrogen atoms see below. Other hydrogen atoms were refined using a riding model starting from calculated positions. Special features for compound **12**: The material crystallizes as a deuteriochloroform monosolvate; the solvent molecule is ordered. Because the crystal was thin and the structure non-centrosymmetric, the data are somewhat weak and limited in number; an extensive system of restraints to the displacement parameters (DELU/SIMU) was used to improve refinement stability. The OH hydrogen atoms were not located in difference syntheses, and were therefore optimized to a suitable hydrogen bond geometry, then the OH groups were allowed to rotate. The positions thus obtained are plausible but should be interpreted with caution. The Flack parameter refined to 0.2(2).

Crystallographic data have been deposited with the Cambridge Crystallographic Data Centre as supplementary publications no. CCDC-1007877 (**12**) and -1007878 (**21**). Copies of the data can be

obtained free of charge from www.ccdc.cam.ac.uk/data_request/cif.

Table 1. Hydrogen bonds [Å and °] for **12**·$CDCl_3$.

D-H···A	d(D-H)	d(H···A)	d(D···A)	<(DHA)
O(2)-H(02)···O(1)#1	0.84	1.98	2.791(8)	161.0
O(4)-H(04)···O(3)#2	0.84	2.13	2.917(8)	156.5
C(7)-H(7)···O(3)#2	0.95	2.36	3.307(10)	177.6
C(22)-H(22B)···Cl(3)#5	0.99	2.78	3.750(9)	167.7
C(99)-D(99) ···O(4)	1.00	2.50	3.460(10)	161.2

Symmetry transformations used to generate equivalent atoms: #1 -*x*+1,-*y*+2,*z*-1/2; #2 -*x*+1/2,*y*,*z*-1/2; #3 -*x*+1,-*y*+1,*z*+1/2; #4 *x*+1/2,-*y*+1,*z*; #5 -*x*+1/2,*y*,*z*+1/2.

Table 2: Crystallographic data for **12**·$CDCl_3$ and **21**.

Compound	**12**·$CDCl_3$	**21**
Formula	$C_{25}H_{24}DCl_3O_4$	$C_{24}H_{20}O_2$
M_r	496.81	340.40
Habit	colourless plate	yellow tablet
Cryst. size (mm)	0.3 × 0.12 × 0.02	0.4 × 0.2 × 0.08
Crystal system	Orthorhombic	monoclinic
Space group	$Pca2_1$	$C2/c$
Temperature (°C)	−140	−140
Cell constants:		
a (Å)	19.286(3)	14.051(2)
b (Å)	12.253(2)	8.0225(12)
c (Å)	9.527(2)	14.536(2)
α (°)	90	90
β (°)	90	96.695(6)
γ (°)	90	90
V (Å^3)	2251.2	1627.5
Z	4	4
D_x (Mg m^{-3})	1.466	1.389
μ (mm^{-1})	0.44	0.09
F(000)	1032	720
$2\theta_{max}$	52.6	60
Refl. measured	8580	9043
Refl. indep.	3249	2394
R_{int}	0.112	0.081

Parameters	413	119
Restraints	293	0
$wR(F^2$, all refl.)	0.165	0.140
$R(F, >4\sigma(F))$	0.091	0.051
S	1.19	1.06
max. $\Delta\rho$ (e Å^{-3})	0.43	0.45

Reference: [1] Sheldrick, G. M. *Acta Cryst*. (**2008**), *A64*, 112-122.
